# Rapid prototyping of multi-compartment models for urea kinetics in hemodialysis: a System Dynamics approach

**DOI:** 10.1007/s10047-023-01416-w

**Published:** 2023-09-05

**Authors:** David M. Rubin, Robyn F. R. Letts, Xriz L. Richards, Shamin Achari, Adam Pantanowitz

**Affiliations:** 1 Biomedical Engineering Research Group, School of Electrical and Information Engineering, University of the Witwatersrand, Johannesburg, 1 Jan Smuts Avenue, Braamfontein, Johannesburg, South Africa; 2 Wits Innovation Centre, University of the Witwatersrand, Johannesburg, 1 Jan Smuts Avenue, Braamfontein, Johannesburg, South Africa

**Keywords:** Hemodialysis, System Dynamics, Modeling, Multi-compartment

## Abstract

Models of urea kinetics facilitate a mechanistic understanding of urea transfer and provide a tool for optimizing dialysis efficacy. Dual-compartment models have largely replaced single-compartment models as they are able to accommodate the urea rebound on the cessation of dialysis. Modeling the kinetics of urea and other molecular species is frequently regarded as a rarefied academic exercise with little relevance at the bedside. We demonstrate the utility of System Dynamics in creating multi-compartment models of urea kinetics by developing a dual-compartment model that is efficient, intuitive, and widely accessible to a range of practitioners. Notwithstanding its simplicity, we show that the System Dynamics model compares favorably with the performance of a more complex volume-average model in terms of calibration to clinical data and parameter estimation. Its intuitive nature, ease of development/modification, and excellent performance with real-world data may make System Dynamics an invaluable tool in widening the accessibility of hemodialysis modeling.

**Supplementary Information:**

The online version contains supplementary material available at 10.1007/s10047-023-01416-w.

## Introduction

Urea is one of many molecules that are cleared by renal function. Its toxicity is uncertain, yet it remains an important indicator of the effectiveness of renal replacement therapy [1, 2]. The single-compartment model for urea distribution, which initially gave rise to the use of *kt*/*V* as a measure of dialysis efficacy, assumes that urea is distributed in a single, well-mixed volume corresponding to the total body water [1].

The observed rebound behavior of urea on cessation of dialysis led to the development of alternative models including the dual-compartment approach, to account for this effect [3]. This approach assumes that urea is distributed between a smaller extracellular volume including the blood plasma and a larger intracellular volume. An inter-compartmental mass transfer coefficient characterizes the diffusion of urea across the membrane separating the two volumes.

Dual-compartment modeling has introduced modifications to the determination and interpretation of *kt*/*V* [3], and an analytical solution to multi-compartment modeling has been developed [4].

While kinetics modeling in dialysis could improve patient outcomes, it is often viewed as a complex, abstract activity, far removed from clinical practice [2]. To make the modeling more accessible to a range of health care workers and trainees, we have developed a System Dynamics (SD) stock-flow model of dual-compartment urea kinetics. The intuitive, graphical nature of SD models makes them easy to construct, adapt, and modify.

The SD stock-flow approach developed by Forrester [5] has been applied to renal modeling [6]. Azar et al. describe an SD model of dialysis efficacy comprising two stocks, namely the post-therapy blood urea nitrogen (BUN) and calculated dialysis adequacy in terms of *kt*/*V* [7]. Their model incorporates many of the factors and policies in hemodialysis administration, but it does not constitute a compartmental pharmacokinetic model per se.

In SD, each stock and its associated flows represent a first-order differential equation which may be linear or non-linear. Thus, a System Dynamics model represents a set of coupled first-order differential equations [5]. Forrester points out that the stock-flow notation reflects the more natural and intuitive integral equation interpretation wherein flows give rise to accumulations (stocks) [5]. This, combined with the graphical nature of the interface, is what makes the SD approach so widely accessible.

The suitability of SD to rapid prototyping and manipulation of models arises because the differential (integral) equations are created graphically by interconnecting the stocks and flows, while the behaviors of these elements are governed by simple algebraic relationships.

To demonstrate the feasibility of rapid compartmental modeling of urea kinetics, we develop a dual-compartment model and compare its performance with the Volume–Average (*V*–*A*) model of Sano et al. [8]. We chose this model for comparison, because Sano et al. present a dual-compartment model based on detailed first-principles, multi-scale analysis which is very different from the lumped SD approach. They achieve an excellent fit with measured urea concentration data, originally from Ono et al. [9], thus facilitating the comparison of our model with theirs. Sano et al. [8] also demonstrate how their model incorporates the urea rebound phenomenon on cessation of dialysis, which as they show, is absent in the models of Gotch et al. [10] and Shinzato et al. [11].

## Materials and methods

### Model construction

**Fig. 1:**
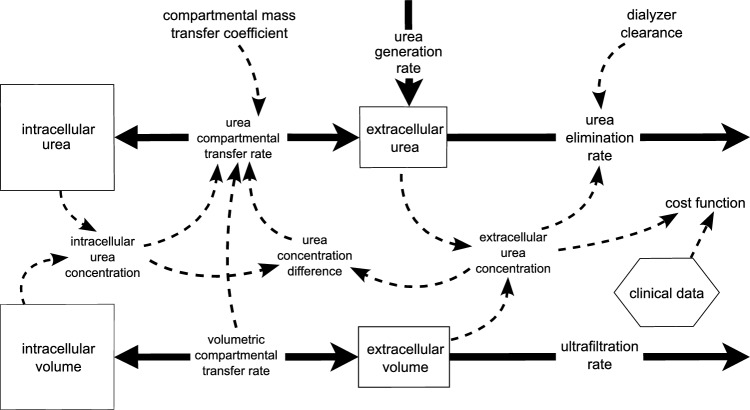
Redrawn System Dynamics stock-flow diagram of the dual-compartment hemodialysis model created in Insight Maker

We constructed a stock-flow model in Insight Maker [12], which is redrawn in Fig. 1, to simulate two-compartment urea kinetics during dialysis. The model comprises four stocks and five flows.

The stocks are: intracellular volume, extracellular volume, intracellular urea, and extracellular urea. The flows are: urea generation rate, inter-compartmental urea transfer rate, urea elimination rate, inter-compartmental volume flow rate, and dialyzer ultrafiltration rate. The intracellular and extracellular urea concentrations are calculated dynamically during model simulation from the above stocks, as shown in Fig. 1.

In keeping with the *V*–*A* model, we examined three variables for parameter estimation, namely: a urea compartmental mass transfer coefficient ($\phi$) expressed as a volumetric clearance in L/h, dialyzer clearance (*K*) in L/h, and urea generation rate (*G*) in mmol/h.

To compare the performance of our SD model with the *V*–*A* model, we used the same constants as Sano et al. [8] (expressed in alternative units), namely: intracellular volume 21.6 L, extracellular volume 14.4 L, inter-compartmental volume flow rate 0.72 L/ h, and dialyzer ultrafiltration rate 1.2 L/h. In our System Dynamics model, we did not include bulk flow of urea with the ultrafiltration.

A converter holds the measured clinical urea data, [$U_\textrm{C}$] [8, 9] and a simple cost function to be minimized during optimization is defined as, $\sum ({[U_\textrm{C}]} - {[U_\textrm{E}]})^2$, where [$U_\textrm{E}$] is our model output for extracellular urea concentration. Insight Maker performs a direct search optimization which is an adaptation of Powell’s method [12].

### Simulation

We digitized [13] the BUN data for the three patients [9] reported in Sano et al. [8], and converted these to urea concentrations in mmol/L. Approval for this secondary use of data was obtained from our institution’s Human Research Ethics Committee (Medical) (Ref. W-CBP-230315-01). We calibrated our model in Insight Maker by minimizing the cost function, to achieve a best-fit parameter estimation.

To remain consistent with the study by Sano et al. [8], we chose to optimize parameters *G*, $\phi$, and *K*, which they term *S*, *Ah*, and *K*, respectively. The simulation was run for a 5 h time horizon with dialysis running for 4 h. Euler’s method with time increments of 0.001 h was used.

We ran a sensitivity analysis in Insight Maker for extracellular urea concentration for each patient by applying a uniform distribution of values for each of the three free parameters spanning the ± 50% range of the optimized values. The 50% and 95% confidence bounds for urea were plotted for each parameter as shown in the Supplementary Material.

## Results

**Fig. 2:**
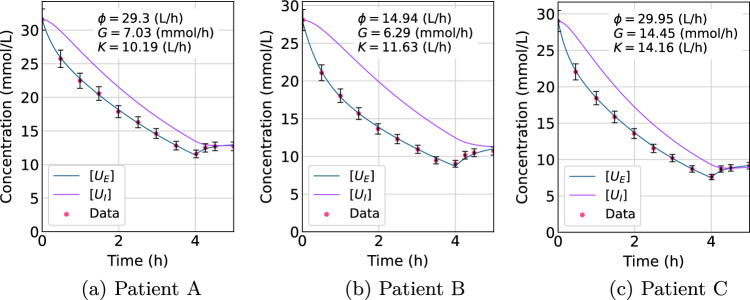
Curves for extracellular $[U_\textrm{E}$], and intracellular $[U_\textrm{I}]$ urea concentrations (mmol/L) vs time (h), fitted by the SD model to extracellular urea concentration data from patients A, B, and C [8, 9]. Error bars of ± 5% are included for each data point [14]. Optimized parameter estimates from this model, $\phi$, *G*, and *K*, are shown for each patient

Simulation of our calibrated SD model using the measured data [8, 9] produced an excellent fit of the model-generated extracellular urea concentration, $[U_\textrm{E}]$, to the measured data for all three datasets, as shown in Fig. 2. Calculated intracellular urea concentrations, $[U_\textrm{I}]$, are also displayed.

While the data [8, 9] did not include uncertainty estimates, we superimposed ± 5% error bars on the data in the graphs in Fig. 2 based on estimated coefficients of variation for urea measurements [14]. The parameter estimates for *G*, $\phi$, and *K* for our SD model were consistent with the range expected and are shown in the insets of Fig. 2 for each patient. The $[U_\textrm{E}]$ curves produced by the SD model display a good fit with the data. Slight overshoot after termination of dialysis (4–5 h) is evident for patient C, however, the fitted curve lies within the ± 5% error bars for every data point, indicating that the fit is consistent with laboratory uncertainty.

A tabulated comparison of our optimized parameter estimates, *G*, $\phi$, and *K*, for the SD model with those in the *V*–*A* model termed *S*, *Ah*, and *K*, respectively, is presented in the supplementary material.

Sensitivity curves generated by our SD model for each of the parameter estimates, and for each patient, are shown in the supplementary material. The sensitivities were analyzed for a uniform distribution of parameter values with a ± 50% variation around the optimum estimated parameter and the 50% and 95% confidence limits are shown in each case.

It is evident from the sensitivity curves that of the three parameters, the extracellular urea concentration is least sensitive to variations in urea generation rate, *G*, and compartmental mass transfer coefficient, $\phi$, and most sensitive to variations in dialyzer clearance, *K*.

## Discussion

In addition to predicting dialysis adequacy, modeling may help practitioners to understand the underlying mechanism of solute and fluid transfers.

Given the urea rebound on cessation of dialysis, dual-compartment models achieve better correspondence with measured urea concentrations than single-compartment models. However, they remain imperfect. For example, Schneditz et al. [15] suggest that the apparent mass transfer coefficient between the intracellular and extracellular compartments may, in fact, represent a perfusion term. If this proves to be so, it would require modification of our mechanistic understanding, and therefore also our models.

Model architecture and the choice of constants can substantially influence behavior. In our SD model, we used the same numerical values for volumes, ultrafiltration rate constant, and inter-compartmental volume flow rate as the *V*–*A* model to facilitate direct comparison. In future work, these values may be reconsidered based on new findings. For example, Sano et al. argue that the inter-compartmental volume flow rate is roughly constant during dialysis; however, these assumptions need to be reviewed. As mentioned, we did not include urea bulk flow due to ultrafiltration. The extent to which these choices are reasonable will depend on more detailed physiological data. However, the ease with which these modifications can be accommodated in an SD model is evident.

The optimized best-fit parameter values for $\phi$, *K*, and *G* are shown in each panel of Fig. 2, and are compared with the values obtained in the *V*–*A* model called *Ah*, *K*, and *S*, respectively, as shown in the supplementary material. These parameters are known to be difficult to determine, and various models produce widely different estimates [8, 9]. However, the difference in parameter estimations between our SD model and the *V*–*A* model being greatest for urea generation rate, *G*, and compartmental mass transfer coefficient $\phi$, is consistent with the very low sensitivity of extracellular urea concentration to the parameters *G* and $\phi$ (see supplementary material), which allows for a wide range of these parameters while still achieving a good fit to the data. In contrast to this, the closest match between the SD model and the *V*–*A* model was in dialyzer clearance, *K*, and this is consistent with the very high sensitivity of extracellular urea concentration to *K*, as shown in the supplementary material.

## Conclusion

For a hemodialysis model to be useful, it should be mechanistically consistent with known physiology, simple to use, and have strong predictive power for extracellular urea concentration.

The dual-compartment SD model presented in this study, when used with the same constants and clinical data as the *V*–*A* model, produces a comparable fit, which is not surprising given the latitude provided by three free parameters. The SD model does produce slight extracellular urea overshoot in patient C during the post-dialysis phase which is well within the ± 5% uncertainty of clinical measurement of urea [14], indicating that the model is able to describe two-compartment urea kinetics with the requisite accuracy. As shown in the sensitivity analysis presented in the supplementary material, the model is very insensitive to *G* during dialysis which is expected as the urea generation rate is very small compared to rapid urea removal by the dialyzer. On cessation of dialysis, there is no further removal of urea, and thus, the model becomes more sensitive to *G* which may account for the slight overshoot in patient C.

The values for the optimized dialyzer clearance, *K*, between the two models are remarkably close, and the optimized parameter values for compartmental mass transfer coefficients, $\phi$, and urea generation rates, *G*, in both models are consistent with ranges described in the literature [15–19]. These parameters are difficult to estimate [18, 19], and there is no basis for favoring the estimates from either model.

The SD and *V*–*A* models exhibit comparable performance for dual-compartment urea kinetics in hemodialysis. However, SD models can be readily modified and adapted based on new insights, making it suitable for use by physicians, undergraduates, and specialist trainees. We advocate for the use of System Dynamics modeling in renal hemodialysis as a means of enhancing the mechanistic understanding of solute behavior, with the flexibility of rapid model prototyping in a highly intuitive manner.

### Supplementary Information

.

Supplementary file 1 (pdf 1842 KB)

## Data Availability

The numerical constants and mathematical expressions used to populate the SD model will be made available on email request.
