# Supplementary material for: Rapid prototyping of multi-compartment models for urea kinetics in hemodialysis: a System Dynamics approach
Source: J Artif Organs. 2023 Sep 5;27(3):316–20. doi: 10.1007/s10047-023-01416-w (PMC11345323; doi:10.1007/s10047-023-01416-w)
Supplement: Supplementary file 1 — (pdf 1842 KB) [file 10047_2023_1416_MOESM1_ESM.pdf]

SUPPLEMENTARY MATERIAL

Rapid prototyping of multi-compartment models for urea kinetics in hemodialysis: a System Dynamics approach

David M. Rubin, Robyn F.R. Letts, Xriz L. Richards, Shamin Achari, Adam Pantanowitz

Parameter estimation

A parameter estimation was performed on the SD model in Insight Maker by minimizing the sum of squares of the difference between the clinically measured and the calculated extracellular urea. The parameters are: compartmental mass transfer coefficient, ϕ, the urea generation rate, G, and the dialyzer clearance, K. Representative parameters are shown in Table 1 alongside the equivalent parameter estimates from the V-A model of Sano et al. [8], namely: Ah, S, and K. Data from the V-A model have been converted to units of L/h and mmol/h.

Table 1: **Representative parameter estimations of ϕ, G, and K of our SD model compared to the equivalent parameters, Ah, S, and K in the V-A model of Sano et al. [8], expressed in** L/h **and** mmol/h

	SD model			V-A model		
	ϕ (L/h)	G (mmol/h)	K (L/h)	Ah (L/h)	S (mmol/h)	K (L/h)
Patient A	29.30	7.03	10.19	27.0	5.79	10.02
Patient B	14.94	6.29	11.63	16.80	9.64	11.82
Patient C	29.95	14.45	14.16	28.80	13.07	13.62

Sensitivity tests

Sensitivity tests were conducted in Insight Maker on the SD model to establish the sensitivity of the model-generated extracellular urea concentration, $[U_E]$, to variations in parameters K, ϕ, and G. These tests were performed by specifying a uniform distribution of parameter values in the range $\pm 50\%$ of the optimized parameters, ϕ, G, and K, in Table 1. The results are plotted in Figure 1 of this supplement, showing the median, and 50% and 95% confidence intervals (CI) of extracellular urea concentration, $[U_E]$, which results from the parameter distributions specified above. The greatest sensitivity is for K, and the lowest sensitivities are for G, and ϕ.

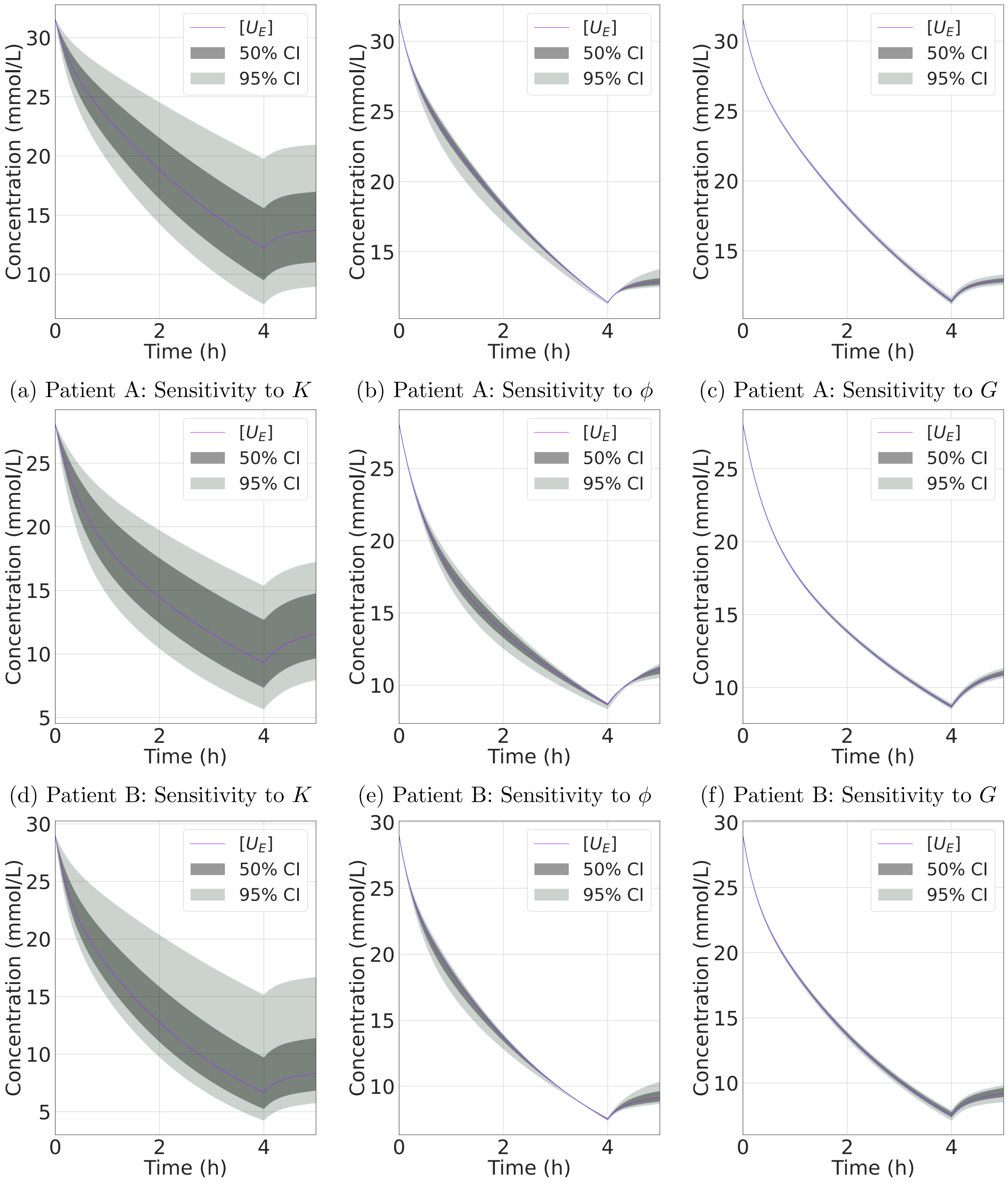

(a) Patient A: Sensitivity to K
(b) Patient A: Sensitivity to ϕ
(c) Patient A: Sensitivity to G
(d) Patient B: Sensitivity to K
(e) Patient B: Sensitivity to ϕ
(f) Patient B: Sensitivity to G
(g) Patient C: Sensitivity to K
(h) Patient C: Sensitivity to ϕ
(i) Patient C: Sensitivity to G

Figure 1: System Dynamics model sensitivity of extracellular urea concentration, $[U_E]$, to variations in parameters K, ϕ and G. Each row represents a patient, A, B and C. Each column represents one of the three parameters. The data was generated by Insight Maker and shows the response to a uniform distribution in the parameter value of $\pm 50\%$ of the optimized value for that patient. The curves show the median response and the 50% and 95% confidence intervals (CI). The greatest sensitivity is to dialyzer clearance, K, and the lowest sensitivities are to G and ϕ
